# Use of Acoustic Emission and Pattern Recognition for Crack Detection of a Large Carbide Anvil

**DOI:** 10.3390/s18020386

**Published:** 2018-01-29

**Authors:** Bin Chen, Yanan Wang, Zhaoli Yan

**Affiliations:** 1 School of Automation, Beijing University of Posts and Telecommunications, Beijing 100876, China; sumrainfn@bupt.edu.cn; 2 Key Laboratory of Noise and Vibration Research, Institute of Acoustics, Chinese Academy of Sciences, Beijing 100190, China; zl_yan@mail.ioa.ac.cn

**Keywords:** crack detection, tungsten carbide anvil, acoustic emission, pattern recognition

## Abstract

Large-volume cubic high-pressure apparatus is commonly used to produce synthetic diamond. Due to the high pressure, high temperature and alternative stresses in practical production, cracks often occur in the carbide anvil, thereby resulting in significant economic losses or even casualties. Conventional methods are unsuitable for crack detection of the carbide anvil. This paper is concerned with acoustic emission-based crack detection of carbide anvils, regarded as a pattern recognition problem; this is achieved using a microphone, with methods including sound pulse detection, feature extraction, feature optimization and classifier design. Through analyzing the characteristics of background noise, the cracked sound pulses are separated accurately from the originally continuous signal. Subsequently, three different kinds of features including a zero-crossing rate, sound pressure levels, and linear prediction cepstrum coefficients are presented for characterizing the cracked sound pulses. The original high-dimensional features are adaptively optimized using principal component analysis. A hybrid framework of a support vector machine with k nearest neighbors is designed to recognize the cracked sound pulses. Finally, experiments are conducted in a practical diamond workshop to validate the feasibility and efficiency of the proposed method.

## 1. Introduction

**Figure 1:**
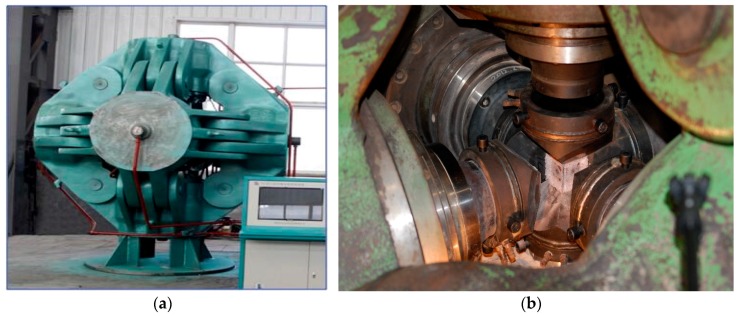
Large-volume cubic high-pressure apparatus. (**a**) Appearance; (**b**) internal anvils.

Synthetic diamond has unique physical and chemical characteristics, such as hardness, semi-conductivity and high thermal conductivity [1]. In China, synthetic diamond is commonly produced by large-volume cubic high-pressure apparatus, which has three pairs of tungsten carbide anvils, as shown in Figure 1. When the apparatus is in operation, it provides 5 GPa pressure and 1500 °C temperature, which is required for the growth of diamond through six hydraulic rams and electric heating mode [2].

Nowadays, the total amount of cubic apparatuses has reached about 6000 in China. Due to the high pressure, high temperature and alternative stresses, the carbide anvil is highly prone to material fatigue. A common form of the failure is cracking, including nucleation, propagation and fragmentation. If the released energy is less than a critical value, the crack does not grow; otherwise, it grows spontaneously. When a cracked anvil is still operating, an unbalance force is exerted on the other five anvils. If not found early enough, it is extremely easy for a cracked carbide anvil to have a serious blowout or even cause casualties. The destruction of tungsten carbide anvils due to blowouts has become one of the most significant economic losses for the manufactures since the apparatuses have a value of 0.05 g/carat.

Traditionally, regular maintenance and subsequent maintenance methods have been applied in synthetic diamond production. During downtime, the anvil is overhauled by observing or sweeping the surface with a saw blade. While in the production process, the currently available detection method is manual monitoring by experienced workers, seriously influenced by strong background noise. In conclusion, these methods lack the ability to accurately judge the state or analyze the health of the carbide anvil, thereby causing poor reliability and inaccuracy. To adequately protect the rest of the anvils prior to a blowout and improve the market competitiveness and production safety, conducting on-line crack detection of the tungsten carbide anvils is necessary.

When a crack occurs in the material, it results in a rapid release of energy, transmitting in the form of an elastic wave, namely acoustic emission (AE). The AE-based detection method has been intensively used in nondestructive assessments of cracks [3,4,5,6,7,8]. Caesarendra et al. proposed an AE-based method for low speed reversible slew bearings, including AE signal processing, feature extraction and pattern classification [3]. Rabiei and Modarres revealed a log-linear relationship between the AE features and crack growth rate, and presented an end-to-end approach for structural health management [4]. Qu et al. presented a comparative study of the damage level diagnostics of gearbox tooth using AE and vibration measurements; the results indicated that vibration signals were easily affected by mechanical resonance, while the AE signals showed a more stable performance [5]. Zhang et al. studied defect detection of rails using AE and wavelet transform at a high speed [7]. In the above methods, the AE sensors are usually attached to the surface of the monitoring object. This is not suitable for crack detection of the carbide anvil because of the high temperature and limited inner space in the apparatus.

More recently, some scholars introduced an AE technique for crack detection of carbide anvils using microphones and have produced in-depth studies on the criterion mechanism of the crack [9,10,11,12]. Han et al. successfully established a tungsten carbide anvil model based on the finite element method (FEM) and indicated that the cracks usually arise around the bevel edge [9]. Li and Wang created a template library of cracking sounds and designed a detection device using voiceprint recognition with an accuracy of 77% [10]. Han et al. combined the Hurst exponent and the neural network to develop a crack detection algorithm of carbide anvils [11]. Subsequently, Yan et al. designed a signal sampling and processing platform based on the digital signal processor and the field-programmable gate array, with a low false-alarm rate of $5.8\times 10^{-4}$ using the sliding time window (STW) technique [12]. This method suffers from the trade-off between the missing-alarm rate and false-alarm rate, with 24 cracked samples misclassified as normal ones among 63 testing samples. The recognition rate is 95% when combined with the STW technique, which takes cost of the real-time performance into account. A more practical crack detection method is still lacking.

Aiming to improve recognition accuracy and generalization, a novel crack detection method based on acoustic emission and pattern recognition is proposed. In this method, the cracked sound pulses are firstly separated from the original signal by preprocessing. According to the mechanism of the crack, three different kinds of features are presented. The high-dimensional features are reduced adaptively by using principal component analysis (PCA). The algorithm combines a k-nearest neighbor (kNN) classifier with a support vector machine (SVM) to refine the classification outcome. Finally, experiments are carried out in a practical synthetic diamond workshop to validate the feasibility and efficiency of the proposed method.

The remainder of the paper is organized as follows. Section 2 presents the principle of the acoustical crack detection method of the carbide anvil based on acoustic emission and pattern recognition. The effectiveness of the proposed method is supported by the experimental work described in Section 3. Finally, conclusions are drawn in Section 4.

## 2. The Proposed Crack Detection Method

When a cracked anvil operates continuously under high-pressure, it generates a typical burst-type AE signal in terms of the sound pulses in time-domain waveform. The sound pulse contains a significant crack information about the anvil. In this paper, crack detection is conducted by recognizing the cracked sound pulses. This is a problem of pattern recognition, consisting of pulse detection, feature extraction, feature optimization and classifier design.

### 2.1. Pulse Detection

#### 2.1.1. Preprocessing

**Figure 2:**
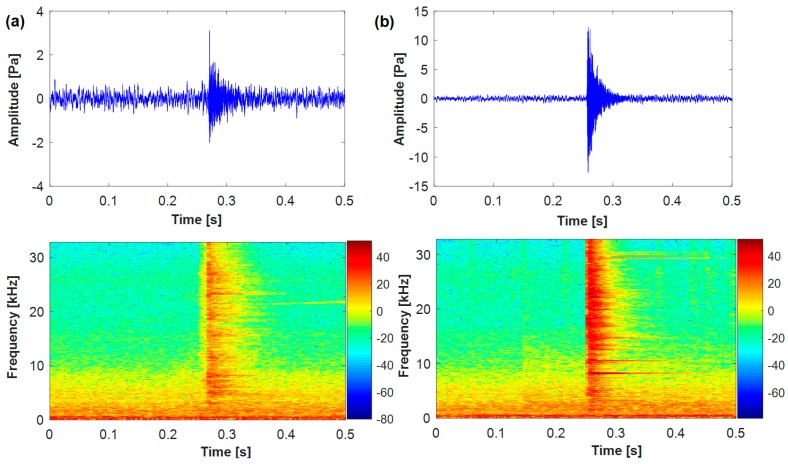
The waveform and time-frequency representation. (**a**) Normal sound pulse; (**b**) cracked sound pulse.

In practical application, there are many normal sound pulses mixed in with background noise, such as knocking, hydraulic cylinder operation noise and so on. Figure 2 shows the waveform and time-frequency presentation for a normal sound pulse and a cracked one. Clearly, the power spectrum of background noise is mainly below 5000 Hz, while the normal and cracked sound pulses distribute in higher frequency, reaching more than 32,000 Hz. The duration of a cracked sound pulse is much shorter, lasting less than 64 millisecond (ms).

When background noise is strong, a small cracked sound pulse is too weak to be detected. According to the spectral characteristic of background noise, a high-pass filter is designed to remove the high-energy low-frequency components in the measuring signal.

#### 2.1.2. Extract Valid Sound Pulses

The high-pass filter makes the small sound pulse more visible. However, there still exists residual noise, which has slight effects on the location of the sound pulse. To find the start and end coordinates of the sound pulses accurately, the threshold of residual noise should be estimated.

Firstly, randomly select a continuous filtered signal and divide it into equal segments. Some of the segments contain sound pulses, while the others do not. They have a remarkable difference in energy.

Secondly, calculate the energy of all segments and select the segments with the energy below a predefined threshold *Q*. The average energy E of residual noise can be calculated by

(1) $$E=\frac{\sum_{i=1}^{P}\sqrt{\frac{1}{N_{1}}\sum_{j=1}^{N_{1}}X_{i,j}^{2}}}{P}$$

where $N_{1}$ denotes the length of segment; *P* denotes the number of objective segments, $X_{i,j}^{}$ denotes the *i*-th objective segment. The initial threshold of noise is set as T=E+K, where *K* is a positive constant, equaling the minimum energy between the sound pulses and residual noise. Parameters *Q* and *K* are specific to experiments.

In practice, the noise is actually time-variant, which also affects the location of the sound pulse. Thus, a fixed threshold is not suitable; instead, a real-time renewal mechanism is designed. When the average energy of new signal $F_{new}$ is greater than the previous threshold *T*, it remains constant; otherwise, it is updated by Equation (2).

(2) $$T=\alpha E{}^{\prime }+(1-\alpha )F_{new}+K$$

where $E{}^{\prime }$ denotes the average energy of the previous noise; α is a weighting coefficient. 

Finally, locate the start and end coordinates of cracked sound pulses. Additionally, the cracked sound pulses must satisfy the following: (a) the average energy of each segment is greater than the threshold T; (b) the duration ranges from 16 ms to 64 ms.

### 2.2. Feature Extraction and Optimization

During diamond production, a large number of normal sound pulses are generated, especially in the process of pressurization and decompression. Some of the normal sound pulses are similar to the cracked ones, as shown in Figure 2. The slight differences, in terms of cracked features, can be found by analyzing the AE signal.

#### 2.2.1. Cracked Feature Extraction

Compared to many of the normal sound pulses, the cracked ones have a larger amount of energy in a high frequency. The zero-crossing rate (*ZCR*) is able to characterize the frequency distribution. The high *ZCR* implies a great proportion of high-frequency components in the signal [13]. For a given signal *x*, the *ZCR* equals the number of times that the amplitude passes through zero [14], defined by

(3) $$ZCR=\frac{1}{2}\sum_{m=0}^{N_{2}-1}\left|\mathrm{sgn}\left[x(m)\right]-\mathrm{sgn}\left[x(m-1)\right]\right|$$

where $m=1,2,\ldots ,N_{2}$, *N*_2_ denotes the length of the signal; the sign function is $\mathrm{sgn}[x]=\left\{ \begin{array}{ll} 1 & x\geq 0\\ -1 & x<0 \end{array}\right.$.

**Figure 3:**
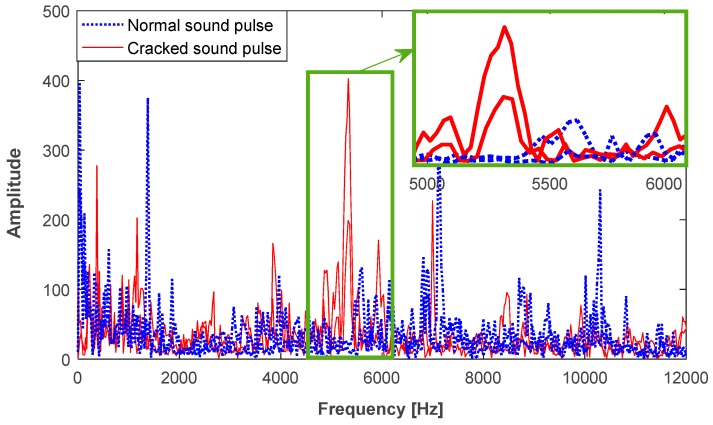
Frequency representation of the cracked sound pulses in combination with normal ones.

Besides, the cracked sound pulses randomly have a sudden change in some frequency bands, as shown in Figure 3. This can be roughly represented in terms of the 1/6 octave frequency band, with a lower frequency, $f_{c}^{lower}=f_{c}/2^{1/12}$, and a higher frequency, $f_{c}^{upper}=2^{1/12}f_{c}$, where $f_{c}$ is the centroid frequency [15]. The sound pressure level (SPL) of the 1/6 octave frequency band is measured in decibels and defined as

(4) $$SPL=20\log_{10}\left(\frac{p_{e}}{p_{ref}}\right)$$

where $p_{e}$ is the measured sound pressure in $\left[f_{c}^{lower},f_{c}^{upper}\right]$; $p_{ref}$ is the reference sound pressure with a value of $2.0\times 10^{-5}$ Pa in air.

The measuring acoustic signal is equivalent to the convolution of the excitation and transfer function. When a crack occurs, the transfer function between the anvil and measuring microphone changes consequently. The linear prediction cepstrum coefficients (LPCCs) stand for the linear prediction coefficients in the cepstrum domain, reflecting the vocal tract by the logarithmic spectrum envelope of the signal in speech recognition [16]. Thus, the LPCC is introduced to remove the excitation, which provides a robust and reliable solution for estimating the transfer function.

In the linear prediction analysis, the current signal is predicted by the linear weighted sum of the past points as [17]

(5) $$\hat{x}(n)=\sum_{i=1}^{R}\beta_{i}x(n-i)$$

where $\beta_{i}$ denotes the prediction coefficients; *R* represents the order of the present prediction. 

The prediction error between the actual and predicted value is given as

(6) $$e(n)=x(n)-\hat{x}(n)$$

The prediction coefficients $\left\{\beta_{i}\right\}$ are determined by minimizing the mean squared error. The LPCCs are derived directly from the linear prediction coefficient, given by

(7) $$c_{n}=\left\{ \begin{array}{l} -\beta_{n}-\sum_{i=1}^{n-1}\left(1-\frac{i}{n}\right)\beta_{i}c_{n-i},1\leq n\leq R\\ -\sum_{i=1}^{R}\left(1-\frac{i}{n}\right)\beta_{i}c_{n-i},n>R \end{array}\right.$$

Finally, the feature vector, consisting of *N* elements of the ZCR, LPCCs and SPLs, is extracted and used to characterize the differences between the cracked sound pulses and normal ones. The feature matrix $B_{M*N}$ is then constructed according to the feature vector, denoted by

(8) $$B={\left[\begin{array}{cccc} b_{11} & b_{12} & \cdots & b_{1N}\\ b_{21} & b_{22} & \cdots & b_{2N}\\ \vdots & \vdots & \ddots & \vdots \\ b_{M1} & b_{M2} & \cdots & b_{MN} \end{array}\right]}_{M\times N}$$

where *M* denotes the number of sound pulses or samples.

#### 2.2.2. Feature Optimization

The redundancy inevitably exists in the feature vector, thereby affecting the accuracy of the classification. Besides, the use of all of the features leads to the problem of high dimensionality and high computational cost. The PCA focuses on a the linear projection of high dimensional data onto low-dimensional subspace by using least-square decomposition while maintaining the maximum variance [18]. This technique is most widely used due to its comparably low computational costs, both in memory and computation time, and its robustness against white noise. Thus, the cracked feature optimization was implemented with the PCA. 

Firstly, calculate the mean-subtracted feature matrix H by centralizing the matrix B as

(9) $$H=\left[\begin{array}{cccc} b_{11}-{\bar{\gamma }}_{1} & b_{12}-{\bar{\gamma }}_{2} & \cdots & b_{1N}-{\bar{\gamma }}_{N}\\ b_{21}-{\bar{\gamma }}_{1} & b_{22}-{\bar{\gamma }}_{2} & \cdots & b_{2N}-{\bar{\gamma }}_{N}\\ \vdots & \vdots & \ddots & \vdots \\ b_{M1}-{\bar{\gamma }}_{1} & b_{M2}-{\bar{\gamma }}_{2} & \cdots & b_{MN}-{\bar{\gamma }}_{N} \end{array}\right]$$

where ${\bar{\gamma }}_{j}=\frac{1}{M}\sum_{i=1}^{M}b_{ij}$, denotes the mean of the *j*-th row.

Then, decompose the feature matrix H with the singular value decomposition (SVD) by H=UΣW, where **U** denotes left eigenvector matrix; W denotes right eigenvector matrix; Σ denotes diagonal matrix. The singular values of matrix Σ in descending order, $\left\{\eta_{l}\right\}$, represent the directions of the variances. The proper number of principal components is indicated by the cumulative contribution rate (CCR), given by

(10) $$\mathrm{CCR}=\sum_{l=1}^{I}\eta_{l}/\sum_{l=1}^{G}\eta_{l}$$

where l=1,2,…,G; G denotes the number of principal components; *I* denotes the number of selected principal components.

The reduced matrix X could be calculated by

(11) $$X_{M\times I}=H_{M\times N}W_{I\times N}^{T}$$

### 2.3. Classifier Design

Crack detection of the anvil is a typical two-class classification problem. The support vector machine has proved to be effective for solving the binary problems and less prone to over fitting [19]. The goal is to find the optimal hyperplane, namely the separating hyperplane, which can separate the data with a maximum margin.

For a given labeled training data set $\left\{\left(x_{1},y_{1}\right),\ldots ,\left(x_{M},y_{M}\right)\right\}$, the basic SVM aims to solve the following optimization problem:

(12) $$\left\{ \begin{array}{ll} \min & F(w,b)=\frac{1}{2}w^{T}w+C\sum_{i=1}^{M}\xi_{i}\\ s.t & y_{i}\left(w\cdot x_{i}+b\right)-1+\xi_{i}\geq 0,\;\xi_{i}\geq 0 \end{array}\right.$$

where $y_{i}\in \{-1,+1\}$ is the class label of the *i*-th training sample $x_{i}$; w is the normal vector of hyperplane; b is a bias. The non-negative slack variable $\xi_{i}$ represents a permitted training error. *C* is a predefined penalty factor, which controls a fraction of the outliers by the trade-off between the training errors and hyperplane complexity.

The decision function is constructed as

(13) f(x)=sgn(w⋅x+b)

Usually, there exist some erroneously classified objects near the separating hyperplane [20]. The kNN technique is used to modify the SVM model. The detailed steps are briefly illustrated as follows.

Step 1: Approximate the posterior probability based on the output of the SVM classifier by a sigmoid calibration function, inspired by Platt [21], denoted as

(14) $$p=\frac{1}{1+\exp(Af+B)}$$

where p∈[0,1]. If the posterior probability *p* is less than 0.5, the corresponding sample is classified as the cracked sound pulse; otherwise, it is normal.

In Equation (14), parameters A and B are determined by solving the following maximum likelihood problem on the training set.

(15) $$\underset{z=(A,B)}{\min}F(z)=-\sum_{i=1}^{M}\left(\frac{1+f_{i}}{2}\log(p_{i})+\frac{1-f_{i}}{2}\log(1-p_{i})\right)$$

Step 2: Find questionable samples from predefined probabilistic interval based on Paüta criterion [22], denoted by

(16) $$\Omega =\left[{\bar{p}}^{2}+\lambda_{2}\sigma_{2},{\bar{p}}^{1}-\lambda_{1}\sigma_{1}\right]$$

where ${\bar{p}}^{1}$ and $\sigma_{1}$ denote the mean and standard deviation of probability for normal sound pulses respectively, while ${\bar{p}}^{2}$ and $\sigma_{2}$ denote the cracked ones; $\lambda_{1}$ and $\lambda_{2}$ are used to adjust the suspicious probability interval, being a positive integer.

Step 3: Calculate distances between questionable samples u and support vectors v, based on kernel function, given by

(17) d(u,v)=k(u,u)−2k(u,v)+k(v,v)

Step 4: Sort the support vectors with the distance in ascending order, and use the class labels of first *k* support vectors to predict the questionable samples.

**Figure 4:**
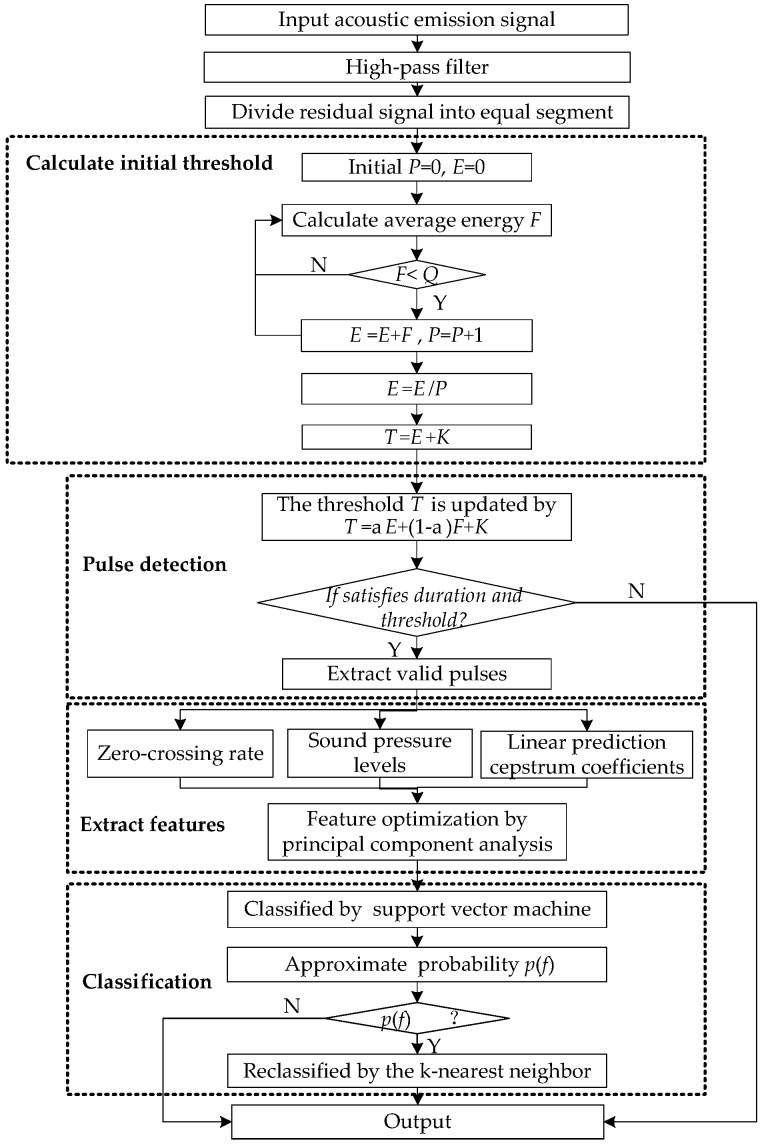
The general block diagram of the proposed method.

The flow chart of the proposed method is shown in Figure 4.

## 3. Experiment and Discussions

**Figure 5:**
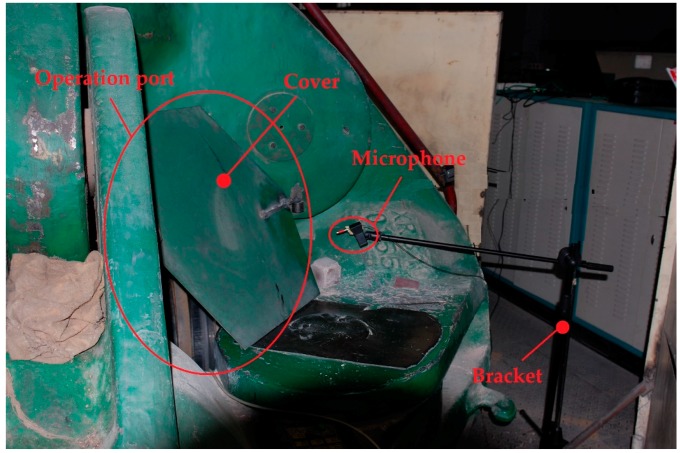
The location of the measuring microphone.

**Figure 6:**
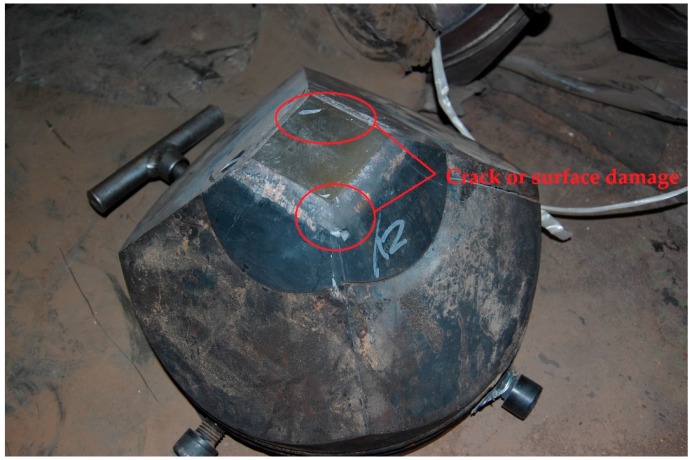
The cracked anvil.

To validate the effectiveness of the proposed method, experiments are conducted in the practical synthetic diamond workshop in Henan Golden Canal Group Co., Ltd. in China. The measuring microphone, B&K 4189, is mounted at one operation port of the apparatus by a bracket, as shown in Figure 5. The acoustic signal is recorded by the B&K Pulse. The normal sound pulses are directly collected from the apparatuses in regular operation, consisting of pressurization, maintaining pressure and decompression pressurization; moreover, these apparatuses continuously work well after a long time. The cracked sound pulses are recorded from experimental apparatus preinstalled on six cracked anvils. Figure 6 shows a cracked anvil with serious surface damage caused by the crack, the location of which is marked by a circular red line. It is noted that the cracked sound pulses are confirmed and labeled by playbacks and discussions with experienced workers.

The choice of sampling frequency is based on the following considerations. In practice, the cracked sound pulses can be distinguished from the normal ones through artificial hearing (20 Hz–20,000 Hz). This means the upper sampling frequency of the measuring signal only needs to be 40,000 Hz. However, the applied B&K Pulse can only adjust the sampling frequency to 8192 Hz, 16,384 Hz, 32,768 Hz, 65,536 Hz or 131,072 Hz. Thus, the 65,536 is finally chosen. The parameters in the sound pulse detection algorithm are listed in Table 1.

**Figure 7:**
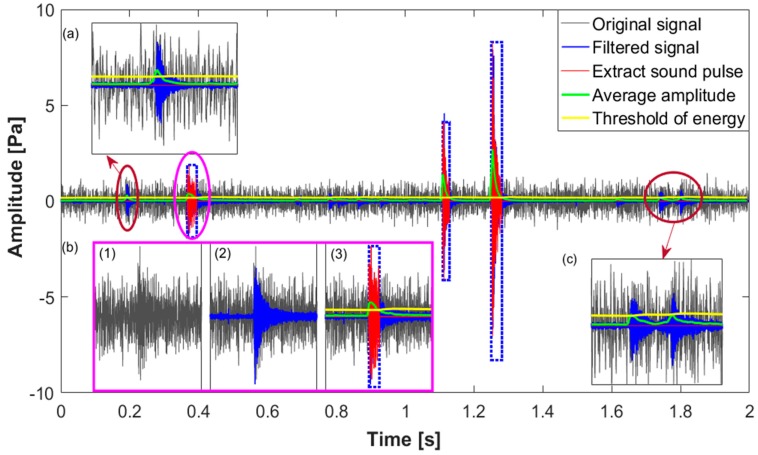
The cracked sound pulse extraction from the original AE signal. (**a**) Normal sound pulse with its duration less than the criteria; (**b**) cracked sound pulse covered up by significant background noise; (**c**) normal sound pulse with its average energy smaller than the threshold.

Figure 7 shows an example of the cracked sound pulse extraction from a continuous signal recording from the apparatus preinstalled on six cracked anvils. Clearly, three cracked sound pulses are located at about 0.35 s, 1.1 s and 1.25 s and are indicated in the blue dashed frame. Compared to the last two pulses, the first one is covered up by significant background noise as shown in subgraph (b); however, it becomes more obvious by using a 5000 Hz high-pass filter. The start and end coordinates are located by comparing the average amplitude and threshold of energy. Besides, some sound pulses are still observed from the filtered signal, as shown in subgraphs (a) and (c). These sound pulses do not match the criteria of duration and average energy as illustrated in Section 2.1, thus they are discarded. This confirms that the proposed method extracts the cracked sound pulses effectively.

**Figure 8:**
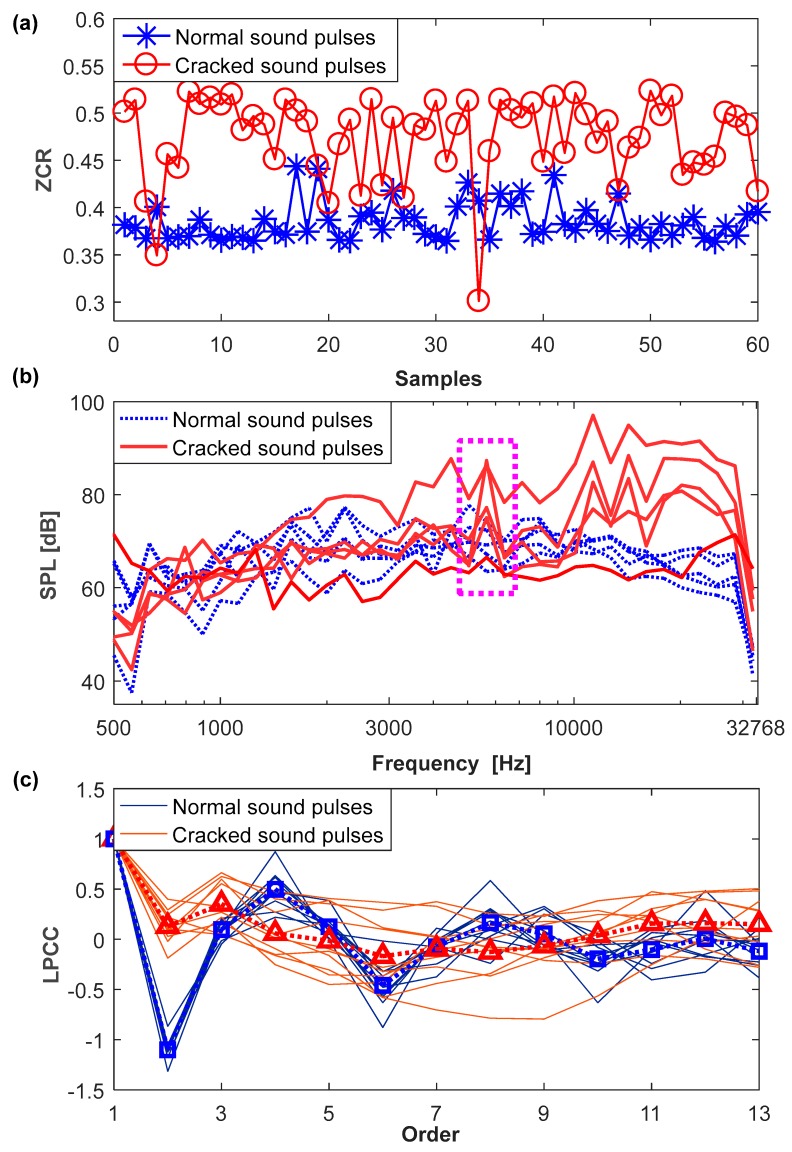
The results of feature extraction. (**a**) ZCR; (**b**) SPLs; (**c**) LPCCs.

In the experiment, 144 cracked sound pulses and 738 normal ones are extracted and used as a data set. Figure 8 shows results of the ZCR, SPLs and LPCCs for some of the cracked sound pulses in combination with the normal ones. 

As shown in Figure 8a, the ZCR of normal sound pulses is stable with the predominant value at about 0.38, while the cracked ones have a greater value, distributed over a larger interval [0.3, 0.55]. This indicates that the cracked sound pulse has more energy in a high frequency band, and the ZCR has an ability to distinguish cracked sound pulses from normal ones. Besides, there is a slight overlap between the normal and cracked sound pulses. With an increase in the number of samples, the overlaps become more serious due to the dispersion and randomness.

Figure 8b shows the SPLs in the 1/6 octave frequency band [500 Hz, 32,768 Hz]. Since the cracked sound pulses may have a short duration of 16 ms, the SPLs below 500 Hz do not exist. Compared to the normal sound pulses, distinct peaks exist in cracked ones, e.g., the 1/6 octave frequency bands [5339 Hz, 5993 Hz], marked by a dashed rectangle. Besides, the SPLs for a majority of the cracked sound pulses have a greater value in the frequency band above 10 kHz. It should be pointed out that this phenomenon does not always exist. In terms of the 1/6 octave frequency band, 35 SPL features are extracted from a sound pulse. 

Figure 8c depicts the results of the thirteenth-order LPCCs, the average value of which is described by marked lines. Obviously, the second-order coefficient for the cracked sound pulses ranges from −0.2 to 0.4, which is different from the normal ones. There are randomly some slight differences at other orders. Since the first-order LPCC has no practical relevance, with a value of one, the other 12 LPCC features are extracted to characterize the sound pulses.

**Figure 9:**
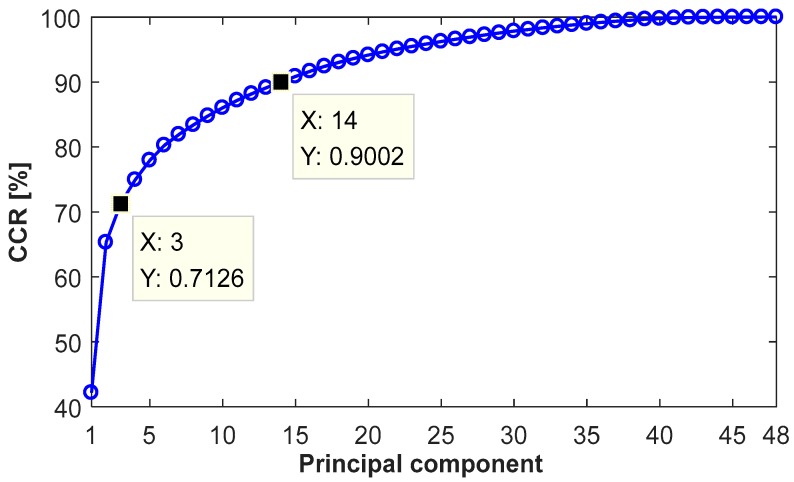
The cumulative contribution rate (CCR) with the increase of the principal components.

Finally, 48 high-dimensional raw features, consisting of ZCR, 35 SPLs and 12 LPCCs, are extracted. Figure 9 plots the results of the feature reduction by the PCA. It can be seen that the CCR significantly increases when the number of principal components rises from one to three, reaching 71.26%. It indicates that three low-dimension principal components contain the most information in sound pulses. Then, the increasing trend gradually flattens due to a reduced amount of information contained in the principal components. When the number increases to 14, the CCR reaches 90.02%. Subsequently, the CCR improves by only 10%, with the number of principal components rising from 14 to 48. A proper number of the principal component is determined by the trade-off between CCR and data simplification. In this way, the feature dimension is reduced significantly.

The selected principal components are used as an input feature vector of the SVM-kNN classifier. In the algorithm, to decrease the false alarm rate, $\lambda_{1}$ is set greater than $\lambda_{2}$ as $\lambda_{1}=5,\;\lambda_{2}=3$; k is set to one-third of the total support vectors; ε is set as 0.5.

**Figure 10:**
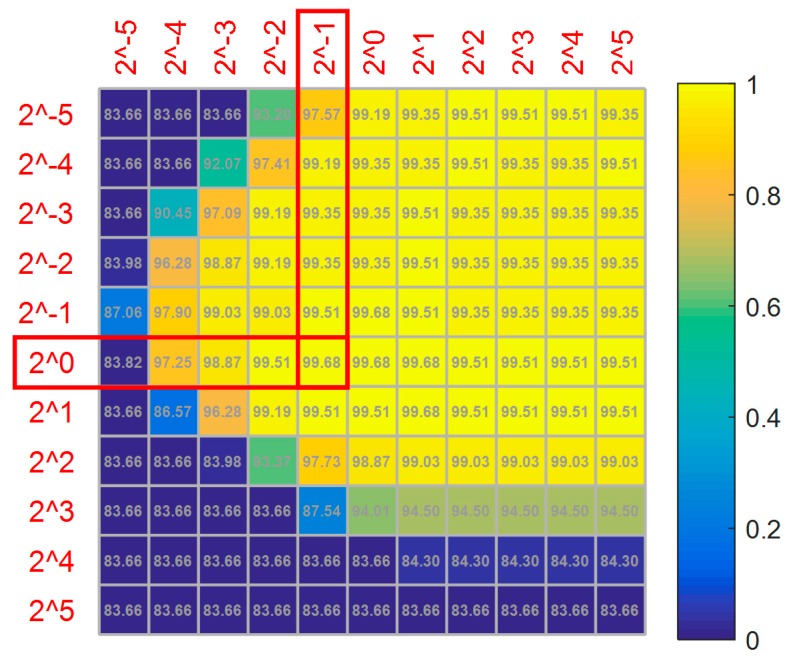
Results of the parameter optimization in the SVM classifier.

The kernel parameter γ of the radial basis function and penalty factor c are optimized by a grid search with a 3-fold cross validation. For example, 70% of the normal and cracked samples is randomly selected as a training set, and the others are selected for testing. Figure 10 gives the optimization results with c as abscissa and γ as ordinate, with the values ranging from $2^{-5}$ to $2^{5}$. The numbers in squares represent the classification accuracy.

It can be seen from Figure 10 that the accuracy reaches a maximum and then decreases with the increase of the penalty factor when the kernel parameter is fixed. It is clear that the best accuracy is 99.68% among 121 (c,γ) pairs. To avoid over-fitting and improve recognition accuracy on the testing set, the minimum pair (0.5, 1) is chosen among five candidate pairs with the same value of 99.68%.

**Figure 11:**
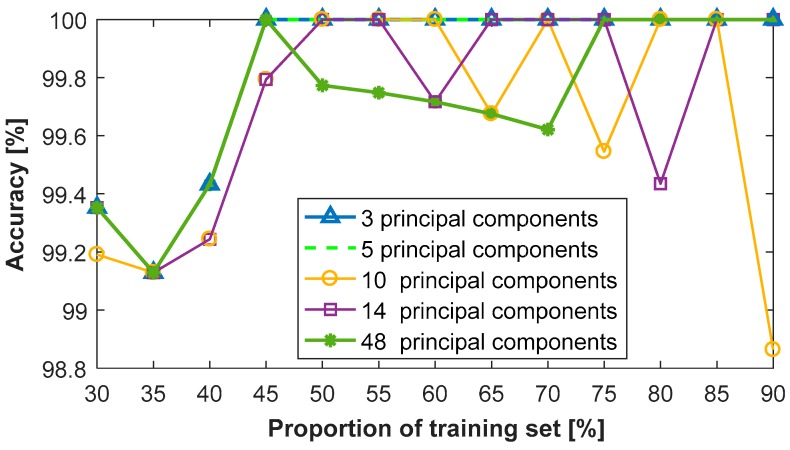
Classification results of the SVM-kNN classifier with different principal components.

Figure 11 shows the classification results using the SVM-kNN classifier with different proportions of the training set and five representative numbers of the principal components, being values of the 3, 5, 10, 14 and 48. Clearly, there is a high accuracy of more than 98.8% for all conditions. The curve of the three principle components overlaps with the one of five principal components. Their accuracies increase rapidly and then remain constant, with an accuracy of 100% with the proportion rising from 45% to 90%. When the number of principal components is 10, 14 or 48, their accuracies slightly decrease randomly after reaching the highest value of 100%. 

**Figure 12:**
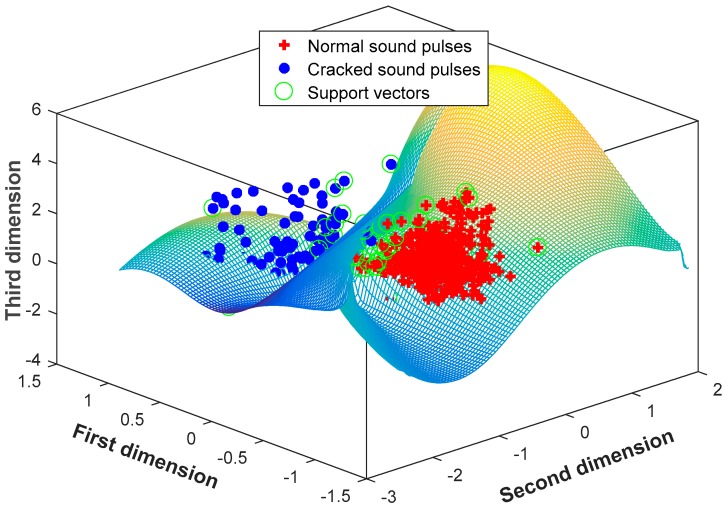
The trained hyperplane with the three principal components.

Obviously, the performance of the SVM-kNN classifier does not improve with an increase of principal components. Taking three principal components as an input feature vector, Figure 12 plots the distribution of training samples, with a proportion of 70%, and the trained classification surface in the three-dimensional coordinate. The number of normal support vectors is 25; the number of cracked support vectors is 26. The normal sound pulses well aggregate, while the cracked ones scatter. They are classified by the hyperplane, with an accuracy of 100%.

**Figure 13:**
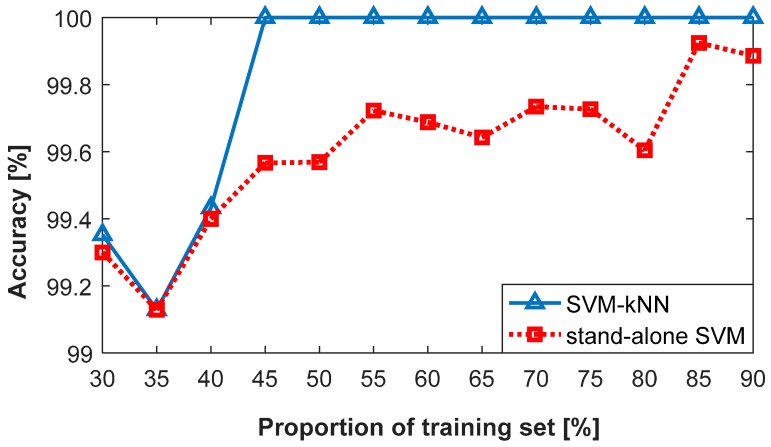
Classification results of the SVM-kNN and stand-alone SVM with the three principal components.

Figure 13 compares the classification results of the proposed SVM-kNN model in combination with stand-alone SVM using three principal components. Clearly, the designed SVM-kNN classifier performs better than the stand-alone SVM; notably, the proportion ranges from 40% to 80%.

## 4. Conclusions

This paper presents an acoustical crack detection method of the carbide anvil based on pattern recognition and the AE signal. By using the noise pretreatment and real-time renewal mechanism, the sound pulse can be separated accurately from the original signal; even the small one becomes more visible. Three kinds of extracted features, ZCR, SPLs and LPCCs, characterize the differences between cracked sound pulses and the normal ones. Feature optimization not only reduces the computation complexity but also obtains a high classification accuracy. The designed hybrid SVM-kNN classifier has a better performance than the stand-alone SVM. The proposed method is verified by the experimental data in a practical synthetic diamond workshop. It is found that the proposed algorithm is able to significantly recognize the cracked anvil, with an accuracy of more than 99%. 

## Figures and Tables

**Table 1:** Parameters of the sound pulse detection.

Length of Segment *N*_1_	Energy Threshold *Q*	Weight Coefficient α	Constant *K*
256	0.1	0.95	0.15
